# Comparative analysis of the prevalence of histopathological findings between DRESS syndrome (drug rash with eosinophilia and systemic symptoms) and drug-induced maculopapular rash: a cross-sectional study

**DOI:** 10.1016/j.abd.2025.501278

**Published:** 2026-01-27

**Authors:** Ludimila O. Resende, João Avancini, Marcella Soares Pincelli, Rodrigo G. Giannotti, Ana Thereza S. Casolato, Cláudia G. Santi, Marcelo A. Giannotti

**Affiliations:** a Department of Dermatology, Universidade de São Paulo, São Paulo, SP, Brazil; b Degree in Business Administration, Universidade de São Paulo, São Paulo, SP, Brazil; c Department of Pathology, Universidade de São Paulo, São Paulo, SP, Brazil

Dear Editor,

DRESS syndrome (drug reaction with eosinophilia and systemic symptoms) is a serious adverse drug reaction characterized by fever, exanthema, and systemic involvement. Observed in 1930 in patients treated with anticonvulsants, the current term was proposed by Bocquet et al. in 1996, seeking to standardize the nomenclature and facilitate diagnosis. The estimated incidence is 1 case per 1,000 to 10,000 drug exposures, with mortality of up to 20%.^1^, ^2^, ^3^

Its etiopathogenesis involves hypersensitivity to drugs or their metabolites, with aromatic anticonvulsants, antidepressants, sulfonamides, nonsteroidal anti-inflammatory drugs, antibiotics, and allopurinol frequently being implicated.^2^, ^3^ The diagnosis is based on clinical-laboratory criteria, initially proposed by Bocquet et al. and subsequently refined by the European RegiSCAR score, which classifies cases as possible, probable, and definite.^4^

Skin biopsy can aid in diagnosis, although there is no single, specific pattern that clearly differentiates DRESS from other drug eruptions, such as maculopapular exanthema (MPE), which shares similar skin morphology but with less clinical severity.^5^, ^6^, ^7^

Given the scarcity of studies on the histopathological characteristics of DRESS in Latin America, this cross-sectional, retrospective, single-center study was conducted to compare the histopathological findings between confirmed and discarded cases of the syndrome, aiming to determine whether DRESS has specific pathological characteristics that allow it to be histopathologically differentiated from MPE. Forty patients hospitalized between 2008 and 2021 at Hospital das Clínicas, Faculty of Medicine, Universidade de São Paulo, were evaluated, all with clinical suspicion of drug-induced skin reaction and available skin biopsy. The medical records were reviewed for application of the RegiSCAR score, with 20 cases being classified as probable or definite DRESS (RegiSCAR > 3) and 20 as MPE (RegiSCAR ≤ 3). Additionally, a scoring system was proposed for the variables that showed a statistically significant difference between the groups, aiming to evaluate the sensitivity and specificity of the findings in distinguishing between DRESS and MPE.

Histopathological analysis was performed by two experienced dermatopathologists, and the criteria evaluated were parakeratosis, spongiosis, isolated keratinocyte necrosis, vacuolar interface dermatitis, papillary edema, pigmentary incontinence, red blood cell extravasation, eosinophilia, and inflammatory infiltrate.

The criteria were graded as described below:

Parakeratosis: Present or absent.

Spongiosis: Absent; mild (< 2/3 of the epidermis); moderate (> 2/3 and without vesiculation); intense (> 2/3 and with intraepidermal vesicles).

Isolated keratinocyte necrosis: Absent; mild (0-2/field); mild intermediate (2-5/field); moderate intermediate (5-10/field); intense (> 10/field).

Vacuolar interface dermatitis: Absent; mild (focal hydropic changes on ×400); intense (diffuse hydropic changes on ×200).

Papillary edema: Absent; mild (subtle); intense (intense with subepidermal blister formation).

Pigmentary effusion: Absent; mild (rare sparse melanophages); intense (melanophages clustered in the papillary dermis).

Hemorrhage: Absent; mild (restricted to the dermal papilla, evaluated at ×400); intense (hemorrhage extending beyond the dermal papilla, evaluated at ×200).

Eosinophilia: Absent; mild (0-10/field); moderate (10-20/field); intense (> 20/field).

Inflammatory infiltrate: Evaluated for density (absent, mild, moderate, and intense) and composition (lymphohistiocytic, neutrophilic, eosinophilic).

For the statistical analysis of these parameters, Fisher's exact test was used. Differences were considered significant if p < 0.05.

For the analysis of the proposed score, Matthews' correlation coefficient (MCC) was used, which ranges from -1 to +1, and the closer to 1, the more reliable the data obtained.

Regarding the results, a significant association was observed between DRESS and the presence of isolated keratinocyte necrosis, intense vacuolar interface dermatitis, and extensive red blood cell extravasation (all with p < 0.05). The other parameters did not differ between the groups (Table 1).

**Table 1:** Comparative analysis of histopathological findings between the DRESS (n = 20) and drug-induced maculopapular exanthema groups. (n = 20).

**Parakeratosis**							
	Present				p-value (Fisher)	PR (95% CI)	OR (95% CI)
DRESS	5				1.00	1.25 (0.39‒3.99)	0.75 (0.17‒3.33)
Maculopapular exanthema	4						


**Spongiosis**							
	Mild		Moderate		p-value (Fisher)	PR (95% CI)	OR (95% CI)
DRESS	8		5		1.00	0.93 (0.60‒1.43)	1.26 (0.33‒4.73)
Maculopapular exanthema	11		3				

**Isolated keratinocyte necrosis**							
	Mild		Mild and moderate intermediary + Intense		p-value (Fisher)	PR (95% CI)	OR (95% CI)
DRESS	6		11		0.007	2.13 (1.20‒3.75)	0.12 (0.03‒0.54)
Maculopapular exanthema	7		1				

**Interface dermatitis**							
	Absent + Mild		Intense		p-value (Fisher)	PR (95% CI)	OR (95% CI)
DRESS	8		12		0.002	6.00 (1.54‒23.44)	0.07 (0.01‒0.41)
Maculopapular exanthema	18		2				

**Papillary dermis edema**							
	Mild		Intense		p-value (Fisher)	PR (95% CI)	OR (95% CI)
DRESS	6		4		0.523	1.43 (0.68‒3.00)	0.54 (0.15‒1.92)
Maculopapular exanthema	7		0				

**Pigmentary incontinence**							
	Mild		Intense		p-value (Fisher)	PR (95% CI)	OR (95% CI)
DRESS	10		3		1.00	1.00 (0.63‒1.58)	1.00 (0.27‒3.67)
Maculopapular exanthema	13		0				

**Red blood cell extravasation**							
	Absent + Mild		Intense		p-value (Fisher)	PR (95% CI)	OR (95% CI)
DRESS	13		7		0.008	‒	0.05 (0.00‒0.89)
Maculopapular exanthema	20		0				

**Eosinophilia**							
	Mild	Moderate		Intense	p-value (Fisher)	PR (95% CI)	OR (95% CI)
DRESS	7	4		5	0.300	1.33 (0.88‒2.03)	0.38 (0.09‒1.54)
Maculopapular exanthema	9	1		2			

**Inflammatory infiltrate density**							
	Absent + Mild		Moderate + Intense		p-value (Fisher)	PR (95% CI)	OR (95% CI)
DRESS	6		14		0.056	2.00 (1.03‒3.88)	0.23 (0.06‒0.87)
Maculopapular exanthema	13		7				

**Inflammatory infiltrate composition**							
Mean	DRESS				Maculopapular exanthema		
Lymphohistiocytic	87.57				94.75		
Neutrophilic	2.71				1.50		
Eosinophilic	6.14				3.75		

Regarding the proposed score, when ≥ 1 it showed 80% sensitivity and 85% specificity for the diagnosis of DRESS, and ≥ 2 had 100% specificity. The MCC was 0.65 for a 1-point score and 0.58 for a 2-point score (Table 2).

**Table 2:** Proposed scoring system for differentiating between DRESS and drug-induced maculopapular exanthema, based on three criteria: intense interface dermatitis (0 or 1-point), intense red blood cell extravasation (0 or 1-point), and keratinocyte necrosis (0 or 1-point – isolated or 2-points – extensive). The total score ranges from 0 to 4.

**1-point rule**					
	0	≥ 1	Total		
DRESS	4	16	20	Specificity	0.85
Maculopapular exanthema	17	3	20	Sensitivity	0.80
Total	21	19	40	PPV	0.81
				NPV	0.84
				Accuracy	0.83
				MCC	0.65

**2-point rule**					
	0	≥2	Total		
DRESS	10	10	20	Specificity	1.00
Maculopapular exanthema	20	0	20	Sensitivity	0.50
Total	30	10	40	PPV	0.67
				NPV	1.00
				Accuracy	0.75
				MCC	0.58

PPV, Positive Predictive Value; NPV, Negative Predictive Value; MCC, Matthews Correlation Coefficient.

The results obtained in this study corroborate previous findings in the literature that indicate that keratinocyte necrosis and interface lesions are frequent markers in DRESS. Overall, interface dermatitis is the most common histopathological presentation reported in the literature, having been found in more than three-quarters of patients with DRESS in previous studies.^7^

The presence of extravasation of red blood cells, possibly resulting from endothelial damage, also stood out as a discriminatory criterion, reinforcing the hypothesis that the dermal endothelium is a frequent target of DRESS.^5^, ^6^, ^7^, ^8^

Although eosinophilia is an important diagnostic criterion in peripheral blood, eosinophilic tissue infiltration did not show correlation with the severity of the condition, both in the present study and in previous analyses; that is, more severe phenotypes did not show a higher density of eosinophilic tissue infiltrate.^5^

As for spongiosis, described in up to 80% of cases in other publications and correlated with favorable outcomes in DRESS,^7^, ^8^ was not statistically significant in the present sample, which may be attributed to the temporal variability between the appearance of lesions and the biopsy performance, and to the sample size.

The study by Cho et al.^7^ demonstrated that the coexistence of three histological patterns (eczematous, interface dermatitis, and vascular damage) is more common in definitive cases of DRESS, being associated with greater clinical and hematological severity. The observation of multiple patterns was also present in part of the present sample, although it was not formally quantified.

Regarding the study limitations, it is a retrospective study, dependent on the analysis of medical records (which are not always complete); it has a medium sample size, which impacts the statistical analysis; and it has a variation between the time of lesion evolution and the biopsy performance, which affects the histopathological findings.

In conclusion, the present study demonstrated that, when comparing patients with DRESS and MPE, the presence of isolated keratinocyte necrosis, intense vacuolar interface dermatitis, and extensive red blood cell extravasation was significantly higher in the first group. Furthermore, the proposed score has relevant discriminatory potential, but its diagnostic application requires validation in independent cohorts and in different centers.

## ORCID ID

João Avancini: 0000-0003-3038-6373

Marcella Soares Pincelli: 0000-0001-9754-0705

Rodrigo G. Giannotti: 0009-0003-9456-2871

Ana Thereza S. Casolato: 0000-0002-4858-4258

Cláudia G. Santi: 0000-0003-3650-4254

Marcelo A. Giannotti: 0000-0002-8911-6020

## Research data availability

The entire dataset supporting the results of this study was published in this article.

## Financial support

None declared.

## Authors' contributions

Ludimila O. Resende: Collection, analysis, and interpretation of data; Critical review of the literature; drafting and editing of the manuscript; approval of the final version of the manuscript.

João Avancini: Design and planning of the study; Effective participation in research orientation; Intellectual participation in the propaedeutic and/or therapeutic conduct of the studied cases; Approval of the final version of the manuscript.

Marcella Soares Pincelli: Critical review of the manuscript; Interpretation of histopathological data.

Rodrigo G. Giannotti: Statistical analysis; Table preparation.

Ana Thereza S. Casolato: Collection, analysis, and interpretation of data.

Cláudia G. Santi: Approval of the final version of the manuscript; Critical review of the content.

Marcelo A. Giannotti: Effective participation in research orientation; Critical review of the manuscript; Approval of the final version of the manuscript; Interpretation of histopathological data.

## Conflicts of interest

None declared.
